# Retraction: “*Haemophilus influenzae* responds to glucocorticoids used in asthma therapy by modulation of biofilm formation and antibiotic resistance”

**DOI:** 10.15252/emmm.201870001

**Published:** 2018-03-12

## Abstract

Chris S Earl, Teh Wooi Keong, Shi‐qi An, Sarah Murdoch, Yvonne McCarthy, Junkal Garmendia, Joseph Ward, J Maxwell Dow, Liang Yang, George A O'Toole & Robert P Ryan

The above article, published May 20 2015 in *EMBO Molecular Medicine*, has been retracted by agreement between the authors of the study, CSE, TWK, SQA, SM, YMcC, JG, JW, JMD, LY, RPR, the journal Chief Editor and the EMBO Head of Scientific Publications in accordance with the outcomes of independent investigations conducted by the University of Dundee and University College Cork.

GAO'T disagrees with the text of this retraction notice, albeit not with the retraction.

The following issues are noted:

- Table 1 contains clinical data described in the paper as being derived from a cohort of asthma patients. However, the provenance of this data is unclear. Based on the evidence available, the University of Dundee investigation concluded that the majority of the patient cohort is likely to be a subset of a cohort of cystic fibrosis patients reported in *PLoS One* 8(12): e82432 (https://doi.org/10.1371/journal.pone.0082432), although in a number of cases the patient's gender is at odds between the two reports.
- The RNAseq data are unavailable on the European Nucleotide Archive under the reported accession number ERG003569. RNAseq data were uploaded with accession number ERS654066 before publication.
- The paper describes use of both prednisolone and prednisone, yet only the latter was used in the study.

